# Public Health Measures to Address the Impact of Climate Change on Population Health—Proceedings from a Stakeholder Workshop

**DOI:** 10.3390/ijerph192013665

**Published:** 2022-10-21

**Authors:** Samira Barbara Jabakhanji, Stephen Robert Arnold, Kristin Aunan, Matthew Francis Chersich, Kristina Jakobsson, Alice McGushin, Ina Kelly, Niall Roche, Anne Stauffer, Debbi Stanistreet

**Affiliations:** 1 Department of Public Health and Epidemiology, School of Population Health, RCSI University of Medicine and Health Sciences, 123 St Stephen’s Green, D02 YN77 Dublin, Ireland; 2 School of Earth and Environment, University of Leeds, Leeds LS2 9JT, UK; 3 CICERO Center for International Climate Research, 0318 Oslo, Norway; 4 School of Public Health, University of Witwatersrand, Johannesburg 2000, South Africa; 5 School of Public Health and Community Medicine, University of Gothenburg, 405 30 Gothenburg, Sweden; 6 Institute for Global Health, University College London, London WC1E 6BT, UK; 7 Irish Medical Organisation, D02 Y322 Dublin, Ireland; 8 Public Health Medicine Environment and Health Group, Health Service Executive, D08 W2A8 Dublin, Ireland; 9 Centre for Global Health, Trinity College Dublin, D02 PN40 Dublin, Ireland; 10 Health and Environment Alliance, 1210 Brussels, Belgium

**Keywords:** climate change, environment and public health, health policy, health equity, evidence to decision, health communication, health co-benefits, climate mitigation, climate adaptation, health-in-all-policies

## Abstract

Background: The World Health Organization identified climate change as the 21st century’s biggest health threat. This study aimed to identify the current knowledge base, evidence gaps, and implications for climate action and health policymaking to address the health impact of climate change, including in the most underserved groups. Methods: The Horizon-funded project ENBEL (‘Enhancing Belmont Research Action to support EU policy making on climate change and health’) organised a workshop at the 2021-European Public Health conference. Following presentations of mitigation and adaptation strategies, seven international researchers and public health experts participated in a panel discussion linking climate change and health. Two researchers transcribed and thematically analysed the panel discussion recording. Results: Four themes were identified: (1) ‘Evidence is key’ in leading the climate debate, (2) the need for ‘messaging about health for policymaking and behaviour change’ including health co-benefits of climate action, (3) existing ‘inequalities between and within countries’, and (4) ‘insufficient resources and funding’ to implement national health adaptation plans and facilitate evidence generation and climate action, particularly in vulnerable populations. Conclusion: More capacity is needed to monitor health effects and inequities, evaluate adaptation and mitigation interventions, address current under-representations of low- or middle-income countries, and translate research into effective policymaking.

## 1. Introduction

Climate change has led to shifts in weather, ecosystems and human systems that are leading to an increasing health burden [1], and the World Health Organization has named climate change as the biggest threat to human health in the 21st Century [2]. The European Academies’ Science Advisory Council groups the effects of climate change on health as direct, indirect via ecosystem effects, or indirect via societal system effects, pointing out that health effects concern both communicable and non-communicable diseases, including mental health problems [3].

Evidence for links between climate change and health have emerged over the past decades, with an increasing number of studies published that demonstrate adverse health effects from climate hazards [4]. Specifically, the changing climate affects health and wellbeing through exposure to hazards including heat and drought, floods, storms, fire and air pollution, sea-level rise, and changing land cover and ocean chemistry. For example, air pollution and extreme heat exposure have both been shown to increase the incidence of cardio-respiratory diseases and mortality, and worsen birth outcomes [3,5,6,7,8,9,10]. Furthermore, extreme heat with low precipitation increases the risk of wildfires [1], which contribute to intense air pollution [11] in addition to existing indoor and outdoor air pollution from use of fossil fuels and burning of biomass, such as for transport, agriculture, heating and cooking [3]. As extreme heat and air pollution are becoming more present, their additive effects have led to a significant increase in hospitalisation and premature mortality in affected regions [3]. Recent record temperatures in Canada [12] and India [13] demonstrated the fatal effects of extreme weather events linked to climate change.

In addition to direct effects, these hazards have secondary effects on health, such as through increasing levels of food insecurity and undernutrition. For example, as precipitation patterns are also changing, droughts have significantly increased in the past two decades, threatening access to safe drinking water and food for the most underserved populations [1]. Additionally, many of these hazards increase the potential for transmission of water-, air-, food-, and vector-borne pathogens which could lead to a higher prevalence of infections such as with cholera, malaria, dengue, zika virus, and chikungunya virus. Unmet healthcare needs are projected to increase as the consequences of climate change lead to forced migration, which bears a high potential for conflict, civil disturbance, and associated trauma [1,3,14]. Changes in relation to the climate, environment, and health are already challenging the wellbeing of people and are projected to increasingly threaten population health in decades to come [1,14,15,16].

Within the current evidence base, impact studies that demonstrate the effect of climate change on health outcomes are the most common [4,17]. What is heavily understudied, however, are the effects of climate change adaptation and mitigation actions on health [4], socioeconomic vulnerabilities and inequalities, climate change effects on working conditions and workers [18], and varying effects based on gender and age [1], thus limiting the evidence base upon which governments can respond to climate change and its health impact.

Despite this, it has become clear that the consequences of climate change on health and social systems are disproportionately affecting disadvantaged communities and populations due to their higher exposure and vulnerability, thus increasing inequities globally [1,14,19] and beginning to reverse past progress made in public health and sustainable development [1]. For example, effects from heat are disproportionally lowering agricultural productivity in low- or middle-income countries (LMICs), measured against total heat-related productivity losses across countries and sectors. Since 1990, the potential agricultural work hours lost due to heat-related factors has increased in low and medium United Nations-defined human development index (HDI) countries, constituting almost half of all potential work hours lost to heat globally in 2020. These decreases in agricultural activity put many agricultural workers and their families under financial strain, lead to lower food availability in affected regions and threaten to reverse past progress in fighting food and water insecurity [1]. The higher vulnerability to climate change and adverse health effects in LMICs is contrasted by their lower contribution to greenhouse gas (GHG) emissions, lower access to climate change mitigation (e.g., slower decarbonisation and poorer air quality regulation) and lower access to healthcare, as shown by the unequal accessibility to COVID-19 vaccines in 2021 [1]. Overall, however, the evidence linking climate change and health remains geographically and thematically scattered, lacks adequate translation into policymaking, and is often considered in isolation to the health research agenda [1,20,21,22,23].

Whitmee et al. (2015) identify three types of challenges in relation to climate change and health: (1) imagination challenges, which include the failure to prioritise future health over current (economic) gains and lead disadvantaged communities and countries to suffer from climate change most; (2) research and information challenges, which are largely dictated by a lack of funding for transdisciplinary research and that include the failure to address social and environmental determinants of health; and (3) implementation and governance challenges, which lead to delayed actions and responses to threat, especially in light of evidence uncertainties, resulting in a delay of positive effects on health and the environment [24].

Currently, the United Nations Paris Agreement, signed in 2015, is the key global framework aimed at reducing GHG emissions to protect human health. The agreement acknowledges the right to health, as well as the vulnerability of individuals and population groups. With their 17 Sustainable Development Goals (SDGs), to be achieved by 2030, the United Nations have developed a roadmap towards an ecosystem in which human needs and rights align with planetary health. To date, this roadmap has not led global leaders on the path of drastic change that is needed for achieving the Paris Agreement goals [1], and more interdisciplinary cooperation between scientists and decision makers is needed to comprehensively inform policymaking [18,24,25]. Unfortunately, the COVID-19 pandemic has further put many of these goals under threat and significantly delayed progress, due to related mortality and morbidity, strain on health systems, economic slowdown, travel restrictions, school closures, and the need to spend resources on pandemic response [26]. Despite ambitious European plans towards fossil fuel independence in response to war in the Ukraine and the Cost of Living Crisis, many barriers remain worldwide that curb this transition and have potential to increase GHG emissions [27].

Given the background of the available evidence, the 2021 report of the Lancet Countdown highlights the World Health Organization recommendations for a healthy and green recovery from the COVID-19 pandemic, which could further help the achievement of the Paris Agreement goals, minimise health inequities and deliver health co-benefits. Between 2018 and 2020, only 0.3% (equating to USD 14 million) of all multilateral climate change adaptation funding (USD 5.1 billion) was directed at health systems, and 13.6% (USD 697 million) of funding has potential secondary benefits for health. Climate change adaptation funding has increased globally in sectors such as waste and water management, agriculture, or the built environment, all of which are relevant to human health [1]. With the Paris Agreement, high-income countries (HICs) committed to jointly mobilising USD 100 billion per year on adaptation and mitigation in low-income countries (LICs), to acknowledge the low spending in LICs relative to their climate vulnerability, as well as the responsibility of HICs for much of the climate crisis. However, while spending needs likely exceed this amount, HICs fail to provide the full spending [28], leading to growing adaptation gaps across and within countries [14], and, overall, significant health adaptation funding is still needed for a green recovery [1].

To build a link between the available evidence, evidence gaps, and policy action that targets the serious impacts of climate change on health, the Horizon-funded *Enhancing Belmont Research Action to support EU policy making on climate change and health* (ENBEL) project seeks to enhance the impact of research findings to support effective climate action and health protection. ENBEL includes multidisciplinary research and policy partners from Europe and Africa, covering geographic research regions in Europe, the Arctic region, East and Southern African regions, the Asia-Pacific region, and the South and Central Americas. More information about ENBEL can be found on the project website [29]. As part of their work, ENBEL commissioned an expert workshop that included the wider conference audience in the debate on the links between climate change and health, aiming to identify particular actions and needs to inform policymaking and public health action, as well as barriers and facilitators to the success of potential interventions.

Presenting findings from this workshop, the aim of this paper is two-fold: (1) to highlight gaps and study requirements to complement the evidence base on the links between climate change and health, and (2) to identify strategies of how the existing evidence on climate change and health can be applied from a public health perspective, and which barriers and facilitators are crucial to their success.

## 2. Materials and Methods

This study used qualitative thematic analysis of data retrieved from an expert panel discussion, which was part of the ENBEL-commissioned workshop.

### 2.1. Data Collection: Description of the Pre-Conference Workshop

#### Workshop Delivery

The workshop was held as a pre-conference to the fully digital 14th European Public Health Conference, on 10 November 2021. The conference had initially been planned to take place in Dublin, Ireland, but was held online due to COVID-19 related public restrictions. In total, 241 conference delegates participated in the pre-conference workshop, a level of attendance that was only exceeded by one other of the 11 pre-conference events [30].

The workshop was opened with three introductory talks (NR, KA, DS), followed by three researcher presentations (KJ, MFC, SRA) on climate and health interventions and research translation into practice that constituted the first part of the workshop (Appendix A). Details of these presentations have been published elsewhere [9,31,32,33,34,35,36]. In the second part of the workshop, an international panel of three public health experts (AMG, AS, IK) and four of the workshop speakers (DS, KJ, MFC, SRA) discussed a number of questions from within the workshop group and the workshop audience, described in this paper. Questions from within the group were collected prior to the workshop. Audience questions were collected during the workshop through a chat function on the conference platform. The panel discussion was chaired by NR. All speakers and panellists are authors to this study and their affiliations and roles are further detailed in Appendix A.

The 60-min-long panel discussion sought to explore how adaptation and mitigation interventions for climate change and health can be implemented in practice, and the role of public health in ensuring an appropriate response to evidence in both HIC and LMIC settings, as well as various supporting questions from within the workshop group and from the audience (detailed below). After addressing these individual questions, all speakers and panellists concluded on the main points from the workshop, as well as next steps for which future need was identified.

The detailed workshop programme can be found in Appendix A.

### 2.2. Panel Discussion Questions

The panel sought to consider the overarching workshop question, ‘What measures can be instituted over the next ten years to address the impact of climate change on population health with a particular focus on the most underserved groups?’ Additionally, Table 1 presents specific questions that were raised and addressed in the panel discussion. Finally, the panel members were asked to provide final points in relation to either of the workshop questions, before conclusions were drawn.

### 2.3. Analysis

The panel discussion was video recorded and transcribed for thematic analysis by two independent researchers, following V Braun and V Clarke [37]. DS and SBJ independently read the transcript and identified initial codes inductively. In a next step, DS and SBJ independently grouped these codes to identify themes that emerged throughout the panel discussion. Following this, DS and SBJ discussed the independently identified themes and their coding to find agreement on the exact themes and their labels. Finally, SBJ re-read the transcript and reviewed the themes to verify their relevance for the panel discussion data, and to map all statements from the panel discussion to at least one of these themes.

A semantic approach to interpretation was chosen [37] as panellists had been asked to make explicit statements in response to the panel discussion questions. Accordingly, SBJ identified quotes from the transcript to highlight examples and to support the identified themes. Additionally, SBJ synthesised the results and supportive literature to link the findings to lessons for policy action and research. No software was used for analysis.

### 2.4. Validation

The initial transcript, coded transcript and themes were shared with all speakers and panellists to confirm that transcripts were correct and statements understood and interpreted according to the views that speakers and panellists had expressed. Additionally, speakers and panellists were asked to verify the correctness and completeness of the themes. Where specific figures, examples or other details had been mentioned in the panel discussion, speakers and panellists were asked to provide literature to support their statements. All speakers and panellists were included as co-authors and approved the findings as presented in this manuscript. Finally, a summary of the results was presented to 25 members of the larger ENBEL project group during their General Assembly in December 2021, and the full manuscript was read by three senior members of the larger ENBEL project group who had not taken part in the workshop, for their independent reviews.

## 3. Results and Discussion

The overarching themes that developed from the panel discussion were that ‘evidence is key’, ‘messaging about health and behaviour change’ is important to consider, that ‘inequalities both between and within countries’ exist, and that there are ‘insufficient resources and funding’. Additionally, the roles of various stakeholders were highlighted by the panel throughout the discussion. This was initially identified as an additional theme of the panel discussion, but during further analysis and validation we reached agreement that it was an integral aspect of each of the four main themes.

Findings from the panel discussion are described under each theme, and supporting literature is cited throughout. As panellists were in agreement with each other’s contributions and their presentation in this manuscript; we did not attribute statements to individual panel members. Additionally, as the panel discussion identified a lack of evidence translation into policy, we provide implications for policy and research that reflect our findings.

### 3.1. Evidence Is Key

One major finding from the panel discussion is that ‘Evidence is key’. The panel acknowledged that a lot of important evidence already exists, mentioning that a recent literature review had reported an 11-fold increase in peer-reviewed original research publications between 2007 and 2020 in relation to the impact of climate change on health. The panel also highlighted that the steepest increase could be seen in the last two years, partly driven by an increased public health awareness during the COVID-19 pandemic. These numbers were confirmed in the original review study [4]. However, the panel also stressed the need for more evidence, and a requirement to translate existing evidence into effective policies that promote a systematic transformation to better health and climate change mitigation within this decade. They stated that “*a lot of what we are doing is piecemeal, rather than systematic*”, which would make it difficult to build the momentum needed to target the complex problems related to climate change. They added that both relatively isolated ‘piecemeal’ and more systematic approaches would be needed, and that there is a need to bring together and integrate the various individual ideas from different stakeholders and from across geographic regions on how to study, adapt to and mitigate climate change.

As a mechanism for generating new evidence, the panel discussed the importance of monitoring, such as has been demonstrated in the annual Lancet Countdown reports [1,38], and the importance of linking outcomes to the SDGs. Importantly, they added that monitoring should cover all SDGs, to minimise the risk that improving one SDG could create a barrier to reaching other SDGs. Additionally, adaptation and mitigation interventions would require scientific evaluation to show their effects, and how these interventions can be implemented in practice. Notably, not only researchers, but a much wider group of stakeholders would have to partake in the development, implementation and evaluation of interventions, including individuals, organisations and governments. This has also been stated in the recent literature [25]. The panel also related this back to the importance of translating existing evidence into policy in order to mitigate or adapt to the effects of climate change.

As part of this, the panel highlighted the importance of defining what ‘effective’ adaptation and mitigation means, and how we can best monitor it. They acknowledged that metrics for mitigation have improved over time, with frequent reporting of GHG emissions or percentage of renewable vs. fossil fuel energy, as can also be seen from the literature [1,3]. However, they noted the absence of clearly defined and universally accepted metrics to measure the effectiveness of climate change adaptation and resilience strategies. To improve their visibility and thus consideration by policymakers, existing adaptation interventions and strategies would benefit from being labelled as climate change adaptation, including public health interventions not originally designed as such. Currently, according to the panel, indicators of global human development, such as education, life expectancy or per-capita gross national income, are not considered indicators of climate change adaptation despite their relevance. The panel flagged the urgent need to change this, as, “*[it] could take years and decades to be able to say what has worked*”, when investigating effects of adaptation strategies on outcomes. They further emphasised that, in addition to broader, more generic outcomes, adaptation and mitigation interventions need to measure small and context-specific details of the impact that individual actions are having. This would highlight the role and abilities of individuals to help mitigate climate effects.

Moreover, the panel suggested to create more evidence through learning from international examples, such as cycling infrastructures in countries with a well-advanced cycling culture, and applying these to other country settings. In line with recent literature [25], they identified that LMICs produce very limited evidence. For example, as stated in the most recent Lancet Countdown report [39], they mentioned that of 850 research articles and editorials published in 2020, only 6 had first authorship from low-HDI countries. According to the panel, one of the reasons for this research gap is a paucity of research funding, and implementing evidence-based examples from well-researched settings in LMICs could help bridge this gap.

Additionally, to study equity and climate change inequity, the panel mentioned a need for better indicators and more disaggregated data than, for example, merely investigating World Bank income groupings. They supported the approach taken by the 2020 Lancet Countdown report. To measure development, this would group countries by the HDI, combining life expectancy as a proxy indicator for health, years of schooling as a proxy for education, and per-capita gross national income. Use of this approach was confirmed in the literature [38]. Furthermore, the panel highlighted that the Lancet Countdown differentiates each of these indicators by gender. While adding complexity to analyses, this disaggregation would be needed to address the paucity of data at a sub-national level, where many of the inequities would be found and where structural change and effective interventions would be needed most. Disaggregated data would further be important in identifying differential intervention impacts across groups.

The panel also mentioned the need to strengthen the use of ‘legal epidemiology’, to investigate the effects of legislation on health and equity, which they recognise is gaining in popularity as a means of tackling climate change worldwide. The important role of legal epidemiology in identifying cause and distribution of ill-health, and in the structuring of environments and behaviours, has been highlighted in prior research [40]. The panel added that this could relate to interventional legislation, analogous to smoking bans or road traffic legislation, which affect individual behaviour, as well as infrastructure legislation where epidemiologists could take on an enforcement role, thus targeting corporations and governments. A recent example by the panel was the death of a nine-year-old girl in London, which they said was the first death ever acknowledged to be a direct result of air pollution, based on scientific evidence [41]. Furthermore, many laws would be inequitable and thus contribute to increasing inequities, for example the panel mentioned that “*with incidental laws, we need to be looking much more [at] equity*”. Research has previously stressed the importance of investigating incidental laws to identify inequities and social determinants of health [40]; however, incidental laws appear understudied in legal epidemiology [42].

According to the panel, building a repository of local and global mitigation actions, such as the legal action taken against large oil companies, could help fill current evidence gaps, drive investments to address the potential for inequities, and challenge laws that impede climate change mitigation. However, the panel mentioned that policymakers should ideally initiate health protection efforts, and that the legal avenue should be a last resort to hold governments accountable. In all cases, they said, it is crucial that science-based evidence and monitoring remains the basis for legal action, and the main role of public health in legal action would be to provide evidence of current and future health risk. In this endeavour, they stated that public health professionals can work alongside those people who are putting forward legal cases. These would often be students and young people taking action towards corporations endangering their health and future, or holding governments accountable for increasing their climate ambition and climate mitigations, such as in Germany and the Netherlands. Examples from these two countries have been published in the literature [43,44,45,46,47]. Lastly, a need was mentioned to monitor the maintenance of government activities, such as when governments or their legal framework are changing, an example by the panel being the withdrawal of the United Kingdom from the EU.

In summary, there was complete consensus among the seven panellists and the chair that “*[w]e need robust science […] to put health at the centre of the climate debate*”, now that momentum has been built through public awareness and acknowledgement of public health during the COVID-19 pandemic.

### 3.2. Messaging about Health for Policymaking and Behaviour Change

The panel further stipulated that it is not only important to generate high-quality evidence, but that the evidence and resulting recommendations need to be effectively communicated to policymakers and the public, in order to achieve improvements in health and the required behaviour change. According to them, the “*message that climate action is needed for health protection is one that still needs to resonate.*” The panel emphasised that more communications are needed, such as the recent effort from the health community on the COP26 climate conference, where millions of doctors and health professionals from more than 500 organisations and from the World Health Organization called on decision makers to phase out fossil fuel subsidies (published in [48,49]). However, the panel added that policies are often built on political compromise, rather than recommendations from the World Health Organization or science. “*If it was only a matter of the science, our policies would be much further [developed], we know enough [about the relationship of climate change and health]*”.

Additionally, the panel noted that, within the health sector, climate action is often overlooked or postponed due to problems that seem more urgent, such as acute care, waiting lists or the COVID-19 pandemic. As housing problems or traffic might similarly lower the perception of climate change as a top priority in other sectors, integrating climate action into all of these sectors would be very important. Consequently, the panel members agreed that a health-in-all-policies approach is needed, where decision making across sectors includes the health sector [17]. According to the panel, “*this means for us as the health community, [that we need] to leave our comfort zone and talk to people we do not normally talk to [and] increase having exchanges*”. In particular, they said that public health needs to find windows of opportunity, such as COP26 or the increased awareness of public health that now exists due to the COVID-19 pandemic, and to take advantage of decision-making processes at a political level that have the potential to reach the entire population. The panel acknowledged that there is slow improvement in this regard. One of the Lancet Countdown indicators assesses government engagement in health and climate change, which increased according to the panel and the latest Lancet Countdown report [38]. Namely, they reported that, as a result of the COVID-19 pandemic, 91 of 195 heads of state linked climate change and health in their United Nations General Debate speeches in 2020, compared to only 43 in 2019 [38].

To support change within the countries where researchers and public health experts are situated, the panel stressed that experts need to highlight to their national policymakers how to become pioneers and good examples in climate action, both personally and governmentally. For example, the positive co-benefits message was highlighted as being influential by communicating how climate action leads to health benefits. Cited examples from the panel include the promotion of active transport to cut GHG emissions and air pollution, which would naturally lead to increases in physical activity and reductions in respiratory and cardiovascular disease, as well as the promotion of ‘green’ or ‘blue’ spaces to improve air quality and lower urban heat, which has been linked to mental health gains in previous research [25,50,51]. The panel stressed that these co-benefits have the potential to improve the wellbeing of society as a whole, and particularly of the large proportion living in urban environments. As previously reported by the Intergovernmental Panel on Climate Change (IPCC) [51], the ENBEL panel added that climate action tends to be cost-saving, stating that, “*[w]e need to be making the economic argument that prevention pays. We always talk about the cost of climate change, but we should maybe not use the language of cost, it is an investment in our future, and it is an investment in the future of our children and grandchildren*”. The panel viewed this positive narrative as a powerful tool to drive climate action.

In addition to science-based arguments, the panel also raised the importance of “*winning hearts and minds*”, including those of policymakers. This acknowledged the importance of positive storytelling and using memorable pictures and graphics to support these stories. According to the panel, more stories and positive messages, especially on the health co-benefits, are needed.

To further support climate action integration across sectors, the panel stressed that it should not be communicated as the sole responsibility of isolated actors; rather the public acknowledgement of the interconnectedness of conflicts would have the potential to also highlight mutual opportunities for change. The panel suggested that opportunities can rest on examples from the past, where increasing awareness was built of how individual lifestyle decisions can mitigate health or environmental harm, and how this awareness subsequently led to perceptions among the public and policymakers that certain behaviours were unacceptable. Examples mentioned by the panel include indoor smoking and use of seat belts in transport, where social acceptability has changed rapidly in the past few decades. Notably, “*it was health evidence, it was epidemiological evidence, and it was public health evidence that were underpinning the fact that smoking is bad for us*”. Accordingly, the panel concluded, if health was put at the centre of the argument, perceptions of other activities that harm health through climate effects, too, might change quickly. Using research to make strong, robust statements about this thus would have the potential to build awareness of how individual decisions can mitigate this harm.

In order to facilitate lifestyle changes that support climate change mitigation and human health, the panel suggested that these lifestyle changes need to become more attractive, including among more vulnerable groups. Here, the panel suggested the use of behavioural economics and infrastructure changes and incentives, which help individuals, families and communities to “*make the healthy choice the easy choice*”. According to the panel, examples could be to stop subsidising or to tax unhealthy and carbon-emitting foods, and instead subsidise healthier and less carbon-intensive foods to increase their accessibility. Additionally, redesigning supermarkets to present foods beneficial to health and the planet immediately before checkouts (nudging) would increase their visibility and access. Furthermore, food system models would require change, with improved access to local produce, community gardens and farmers markets. Other ideas included the use of advertisements in the media, including on children’s television programmes and in local schools. The panel mentioned that schools in particular offer opportunities to educate children about the benefits of various behaviours, as children can equally be reached across societal groups.

Overall, the important role of the public health community in delivering these messages about health in relation to climate change was stressed by all panellists.

### 3.3. Inequalities between and within Countries

The panel agreed that both between and within countries there are significant inequalities in health, and in the opportunities to pursue a lifestyle that facilitates good human and planetary health.

According to the panel, this is not surprising, as “*[i]ssues that are affecting health […] are different in different parts of the world*”, that is, across geographic regions and population groups. However, in order to promote equity, the panel stated, we need to acknowledge this heterogeneity and be open and transparent about existing inequities to open up opportunities for change.

The panel noted that during the pandemic, inequalities became very visible in relation to health outcomes and vaccine access, which was also stated in the literature [1], and they added that these inequalities appeared to affect the same groups that are severely affected by climate change. Namely, according to the panel and prior research, these are poorer communities, people in LMICs, people of different minority groups, including ethnic minorities, people with lower access to education, and people with pre-existing chronic medical conditions [14]. Within Europe, the panel reported large inequities between Western and Eastern Europe, such as in relation to the health burden from air pollution, where energy poverty would lead households to burn wood, coal and “*essentially anything*” in order to cook or to heat their homes. As the awareness of these inequities has increased during the COVID-19 pandemic, according to the panel, now is a good moment to take action that aims to increase equity.

However, the panel stressed that due to lack of data, less evidence and awareness exists of inequalities at the sub-national level, making it difficult to convince decision makers of the need to act. As an important tool to help fill this evidence gap, the panel supports the Lancet Countdown report suggestion of using the HDI indicators. For example, they noted that the use of gender- and age-disaggregated data could help further quantify heat effects in the ages of 65+ and pregnant women, and identify additional within-country vulnerable groups. Similarly, they mentioned that rural and urban disaggregation can help identify inequities, for example in relation to use of clean fuels and technologies for cooking.

The panel also flagged the need to increase public awareness of inequities, and to start measuring individual actions and their impact on the environment. The same has been noted in the most recent IPCC report [14]. The panel explained that currently, predominantly high-level impacts are being measured. Less data is available that help individuals understand their own impact and how they can change to have a more positive impact. For example, individuals should consider the impact of holding an online workshop on data transfer and individual electricity use for streaming devices. Another example included individual conflicts of interest, such as among employers and funders, which panellists note should always be reported transparently. Often, links between climate change and the industry would appear hidden, impacting the choice to avail of pension funds and other services offered by organisations with investments in the fossil fuel industry. Even with the best intentions, the panel stated that everyone has conflicts of interest, that we use resources unequally, for example by benefiting from higher incomes and pension systems in the Global North, and that we try to protect ourselves from poverty. The lifestyle in the Global North would still be a contemporary ‘first-world’ lifestyle, “*and until we start finding ways of living better, we are just transporting our problems into poorer countries. One of the biggest ways to get equity is for us to pull back and start living a proper rationed life [and] we need to find a positive way of doing that*”.

Furthermore, the panel highlighted that “*there is individual responsibility, but we also need to enable [change]*”, meaning that individual action needs to be met by government support. An overreliance of public health and climate change interventions on individual behaviour change and a need to shift towards more system-level interventions has also been described in recent literature [52]. The panel stressed that this includes the need for targeted public investment, such as through subsidies [14], an example from the panel being the need for facilitation of solar panel installations on private roofs through government provision. Accordingly, the panel advised that crucial questions for policymakers are, where to invest public money, where to give subsidies, and where to take subsidies out. For example, policies to upgrade buildings and support active mobility would have the potential for “*accelerating the shift to renewable energy [and to] tackle energy poverty*”. The panel emphasised the importance of policymakers making these investments, rather than merely providing the regulatory framework which may not be followed by action.

Further, to highlight where government support is needed, panellists thought that legal epidemiology should investigate where climate-related policies and law, particularly incidental laws, consider people in vulnerable populations. The panel mentioned that this could, for example, help prevent an increase in energy poverty through mal-adaptation, an issue also raised in the literature [14], or capture differences in urban and rural life.

To target inequities within resource-poorer LMICs, the panel suggested that relatively basic, low-tech interventions are needed. Specifically, a toolbox approach was suggested for use in small-scale enterprises, where enterprises can choose from a set of intervention elements in the toolbox to maximise relevance, simplicity and effectiveness in their specific context.

Ultimately, they conclude that “[i]t is not just about targets, it is about how we are going to reach those targets, and there needs to be some reality and facts behind these kind of ideas, otherwise we are always going to leave those poorest populations behind”.

### 3.4. Insufficient Resources and Funding

Largely, the panel acknowledged that all of the above problems are, in part, dictated by capacity problems, with insufficient resources and funding being available particularly in LMICs, which is an issue also identified by the IPCC [14]. As an example, the panel mentioned that 47 of 91 World Health Organization countries developed national climate change and health adaptation plans, but that 69% of those 47 countries report insufficient funding as a barrier to implementing these plans, with 25% reporting zero funding. The same numbers have been published in the latest Lancet Countdown report [1]. Additionally, in European Union policy, the panel understood the ambition and funding of adaptation and mitigation efforts to be insufficient because health would not be at the centre of climate change arguments.

At the time of the panel discussion, a lot of advocacy work in relation to climate action was reported to still happen voluntarily, thus being restricted to few available hours. The panel saw this particularly among clinicians and public health experts, whose main roles would involve other foci with already limited capacities. To target this problem, the panel highlighted the need to increase capacity for climate action and health in non-governmental organisations, civil society and decision-making fora, across geographic and sectoral areas. They valued the contribution of individual actions and interventions, such as those interventions presented in Part I of the ENBEL workshop. To reach their full potential, however, these initiatives would need to be connected, and the various global, national, international, regional and local roles of people across sectors and areas would need to work together and build meaningful relationships. Additionally, the panel raised the need to reflect on how effective actions have been so far, and what actions and investments are needed next, in order to work more systematically and efficiently.

According to the panel and supporting literature, in LMICs in particular, a lack of funding and capacity contributes to the under-representation of these countries in climate action and evidence generation [17]. For example, the panel noted that the Lancet Countdown authors are almost entirely from HICs [1]. The panel added that ambitions to change this are challenged by a lack of funding, and lack of “*people there on the ground who have the ability to create the indicators that we require to be able to cover such a geographical range when looking at the entire world and on an annual basis*”. In an attempt to improve this, they mentioned that the Lancet Countdown is opening regional centres in South America, Asia, Small Island Developing States, and West-Africa, some of which had already opened recently or would be due to open in the near future.

The panel also saw a need for higher capacity in legal epidemiology, to address the inequality embedded in interventions and infrastructure legislation, and to “*[challenge] some of the tax laws and so on […] that are causing barriers to climate change*”.

Overall, the panel discussion concluded that you cannot deliver change without funding or finance, which would be the missing building blocks of an effective health system in many countries. The IPCC elaborates on this in a similar vein [51]. Additionally, importantly, the panel added that another crucial resource that is becoming scarcer, is time. Accordingly, the panel pointed out that “*as public health professionals, we still need to deliver the capacity [that is currently lacking]*”, with the idea of integrating climate change in medical curricula as a concrete example to increase capacity over time.

## 4. Implications for Policy and Research

These findings support the following ten principles that policymakers and researchers should act upon to promote human and planetary health in this decade.

One of the key findings from the panel discussion was the need for **more evidence**, and specifically useful evidence. This means that adaptation and mitigation interventions have to be developed, implemented and evaluated thoroughly from the perspectives of various stakeholders including individuals, organisations and governments. In addition, it is important to monitor outcomes. Part of the latter includes the labelling of interventions as climate change interventions, and the definition of metrics, in order to be able to tell what ‘effective’ adaptation and mitigation means. Broad outcomes, such as global human development indicators (education, life expectancy and per-capita gross national income), need to be measured, as well as small, context-specific outcomes. Better indicators and more disaggregated data at subnational level are required (e.g., based on gender and age; rural vs. urban settings) to study equity and climate change inequity and identify corresponding interventions. High-level impacts need to be measured, as well as factors highlighting the impacts of individuals’ and industry’s actions (e.g., choice of pension funds, energy use), to drive informed behaviour change. Moreover, more data from LMICs are needed to fill existing evidence gaps.

The panel discussion also raised the need to **implement and evaluate strategies**. In particular, those strategies that have been proven effective should be implemented in new settings, such as in under-resourced LMICs. The constant evaluation of implemented strategies can further help increase their efficiency and lead to more systematic implementation.

As climate change is progressing fast, the panel stressed that more efforts are urgently needed to **translate evidence** into effective policies. They highlighted that policies are often built on political compromise or regarded lower priority than seemingly more urgent health problems; however, there is no time left to continue this. Accordingly, better linkage of policies and evidence needs to become common practice. This could be achieved through better communication, more frequent dialogues, and infrastructure for effective collaboration, both between policymakers from different sectors, and between policymakers and climate and public health experts.

Furthermore, the panel discussion identified a need to **integrate approaches**. Specifically, small and rather isolated ‘piecemeal’ approaches need to be integrated with more systematic approaches, and ideas from different stakeholders and geographic regions have to be brought together. Additionally, approaches should be integrated with the SDGs. Ultimately, through highlighting the interconnectedness of health and climate change on various levels, mutual opportunities for change will also be highlighted. This can be an effective mechanism to build awareness of how individual decisions can mitigate harm, and to build meaningful relationships that foster mutual climate action.

Where decision makers may fail to protect human health and the planet, the panel discussion raised the need to strengthen the use of **legal epidemiology**. Based on science-based evidence and monitoring, legal epidemiology should seek to identify current and future health risks. This includes its role in removing barriers to mitigation, identifying and removing inequities, and monitoring maintenance of government activities. A specific example was the panel’s suggestion to build a repository of cases of local and global legal action.

The panel further pointed out the need to **communicate evidence** on the links between climate change and health effectively, both to the public and to policymakers. This includes a need for more positive messages about the health co-benefits of climate action, and about cost-savings and long-term benefits associated with investments in climate action. The use of storytelling, memorable pictures and graphics constitute further tools for effective communication. Through positive examples, policymakers should be shown how they can become pioneers and good examples in climate action, personally and governmentally. In all of this communication, the panel saw a need for more transparency to facilitate and encourage evidence-based behaviour change.

Another major finding in the panel discussion was the need to **identify, acknowledge and target inequities** that exist between and within countries, in relation to health, climate change, climate action and factors that contribute to either. Disadvantages are often seen in poorer communities, LMICs, ethnic and other minority groups, people with less access to education and people with pre-existing chronic medical conditions. More research is needed that identifies and targets existing inequities within and beyond these groups. For example, more evidence should be generated in LMICs to address their under-representation in research. In resource-poorer settings, relatively basic, low-tech interventions have potential to lead to effective adaptation at low cost, to help prevent further widening of inequalities. Moreover, as previously mentioned, legal epidemiology has an important role in identifying the implications of existing policies on different population groups.

The panel discussion also emphasised the need to **combine top-down (policy) and bottom-up (individual)** climate-related health promotion. This means that a variety of stakeholders need to be consulted, including non-governmental organisations, civil society and decision-making fora. At a policy-level, health needs to be put at the centre of the climate change debate, and a health-in-all-policies approach needs to be pursued. National climate change and health adaptation plans need to be developed, and importantly, these also have to be fully implemented. Furthermore, government support is needed to meet and reinforce individual actions. This requires governments to provide both the regulatory framework, and investments (e.g., subsidies for shifting to sources of renewable energy). The panel saw a clear need to make the healthy choice the easy choice, that is, to make climate- and health-friendly lifestyle changes more attractive across societal groups. Behavioural economics, infrastructure changes and incentives offer useful tools to achieve this. Education settings, too, present as a major opportunity here, especially as schools can reach children and their families across societal groups.

In order to facilitate the above recommendations, the panel discussion concluded that there is an urgent need to **increase capacity**, mainly in relation to human capital (e.g., researchers conducting fieldwork) and funding. Without doubt, there is a need for increasing capacity to generate useful evidence, to implement and evaluate strategies, to translate evidence into policies, to integrate and connect existing approaches and stakeholders, to strengthen legal epidemiology, to communicate evidence transparently and effectively, to identify and target inequities, and to promote health and climate action both top-down and bottom-up. Importantly, it is crucial to increase these capacities across geographic and sectoral areas.

Lastly, as long as the above issues persist, the panel discussion led to the realisation that there is a strong need for finding and making use of **windows of opportunity**, at political level and where public attention lies.

## 5. Conclusions

The panel discussion provided an insightful overview of where science and policy stand in relation to climate change and health, demonstrating an increasing level of available evidence and public awareness, as well as evidence gaps and an urgent need to implement, evaluate, translate and integrate research and policies. Notably, little evidence exists to allow comprehensive analysis and addressing of the existing and very serious inequities. Policies do not make sufficient use of existing evidence, especially on health co-benefits of climate action, to prevent further deterioration in climate change, health and related inequalities. Going forward, a global focus on inequalities is needed for all public health intervention decisions which should include both benefits of action and costs of inaction in monetary and other terms.

Public health is well situated to advocate for policy change, so that climate change and its harmful effects on health can be reduced. Communication here is vital, to outline the positive health co-benefits of climate action, supporting a health-in-all-policies approach and linking climate action to the SDGs. Currently, too few resources and too little funding is available to sufficiently put these necessary steps into action. This is a major barrier that needs to be addressed at national and international level, particularly in LMICs.

## Figures and Tables

**Table 1:** Specific questions addressed during the workshop’s panel discussion.

Question
What would you say are the most important factors for us to consider when planning public health responses to climate change evidence?
What is the role of legal action in addressing the impact of climate change and health? Do you see opportunities for collaboration? What is the role of public health practitioners and researchers in this area?
What do we need to do in terms of public health to address inequity? What are key priorities to address the inequity question?
From the heat and work example, it is clear that large corporations and employers have substantial responsibilities in relation to ensuring safe working practices. Whilst there might be some protection for those in the EU, what can be done to promote safe working practices in lower income countries where there is piece work, child labour, etc?
[When mentioning] Conflict of Interest in our journal articles, should we disclose investments in fossil fuels [through our pension funds, for example, of the organisations we are employed by]?
What is the role of public health in ensuring an appropriate response to such evidence in both high- and low-income country settings?
How do we [influence] behaviour change in the ten years that we have, e.g., how do we [decision-makers] achieve that in relation to diet?

## Data Availability

The workshop recording is no longer available to re-watch online.

## Appendix A. Conference Programme and Abstract

The conference programme was first published on the European Public Health conference website and adapted by the authors. The original publication can be found here:

*European Public Health Conference*, 2021. Pre-conference 14th EPH Conference Wednesday 10 November 2021, 09:00–12:40 CET—Public Health Measures to address the impact of climate change on population health. Retrieved 1st June 2022, from: https://ephconference.eu/repository/conference/2021/Preconferences/ENBEL%20EPH%20conference%20programme.pdf.

### ENBEL Workshop—European Public Health Conference 10th November 2021: Public Health Measures to Address the Impact of Climate Change on Population Health

Worldwide, changes in relation to the climate, environment and health are already challenging the wellbeing of people, and are projected to increasingly threaten population health in decades to come. Research is now emerging that clearly demonstrates the links between climate change and population health; however this research is often fragmented or considered in isolation, and translation of findings into policy is lacking. The international ENBEL project aims to collate, co-produce and communicate knowledge in this area and connect climate change and health policy makers, researchers and other stakeholders and target groups, including in low- and middle-income countries.

This ENBEL workshop brings together researchers and public health experts to identify how best to translate the findings of climate and health research into international public health practice. In simple terms, the question we will be seeking to address ‘What measures can be instituted over the next ten years to address the impact of climate change on population health with a particular focus on the most underserved groups?’

After an introduction to the workshop by Niall Roche, Prof. Kristin Aunan will introduce the ENBEL project and Dr Debbi Stanistreet will present the public health perspective on translating evidence into practice. Following from this, three thematic presentations will summarise the current evidence on how specific aspects of climate change impact on health using the Grade Evidence to Decision making framework, and discuss implications of those findings for public health policy and practice. Specifically, Prof. Kristina Jakobsson will discuss heat and work, Prof. Steve Arnold will present on air quality and climate change and finally Prof. Matthew Chersich will elaborate on maternal health and environmental heat.

In the second part of the workshop, Dr Ina Kelly, Anne Stauffer and Dr Alice McGushin will join for a panel discussion to discuss the evidence-based adaptive measures that can be incorporated into national and local public health planning. The panel will discuss the barriers and facilitators that exist, how interventions can be implemented in practice and the role of public health in ensuring an appropriate response to such evidence in both high- and low-income country settings.

Finally, the panel alongside the presenters, will jointly formulate conclusions and outline the future direction of public health measures to address the impact of climate change on population health.

**Time**	**Presentation**	**Speaker (Country)**	**Affiliation**
	**Part 1**		
09.15	Welcome and introduction	Mr Niall Roche (Ireland)	Centre for Global Health, Trinity College Dublin
09.30	The ENBEL project—inking research to practice	Prof. Kristin Aunan (Norway)	CICERO Center for International Climate Research
09.45	From evidence to practice; a public health perspective	Dr Debbi Stanistreet (Ireland)	RCSI University of Medicine and Health Sciences
	**Climate change: from research findings to practice**		
10.00	(a) Heat and work	Prof. Kristina Jakobsson (Sweden)	School of Public Health and Community Medicine, Institute of Medicine, University of Gothenburg
10.20	(b) Air quality and climate change	Prof. Steve Arnold (UK)	Institute for Climate & Atmospheric Science, School of Earth & Environment, University of Leeds
10.40	(c) Maternal health and environmental heat	Prof. Matthew Chersich (South Africa)	University of the Witwatersrand, Johannesburg
11.00	Coffee break		
	**Part 2**		
11.15	Panel discussion of How such interventions can be implemented in practice: The role of public health in ensuring an appropriate response to such evidence in both high- and low-income country settings	Dr Ina Kelly (Ireland)	President of the Irish Medical Association/Consultant in Public Health medicine/Chair of the HSE’s Public Health Medicine Environment and Health Group
		Ms Anne Stauffer (Europe)	Deputy Director and Head of Strategy for Health and Environment Alliance Europe
		Dr Alice McGushin (UK)	Programme manager for The Lancet Countdown
12.15	Conclusion and next steps	All	
12.30	Close	Mr Niall Roche (Ireland)	Centre for Global Health, Trinity College Dublin
